# Editorial: Theory of mind in relation to other cognitive abilities

**DOI:** 10.3389/fpsyg.2022.1123321

**Published:** 2023-01-18

**Authors:** Ann Dowker, Douglas Frye, Hiromi Tsuji

**Affiliations:** ^1^Department of Experimental Psychology, Oxford University, Oxford, United Kingdom; ^2^Graduate School of Education, University of Pennsylvania, Philadelphia, PA, United States; ^3^Department of Psychology, Osaka Shoin Women's University, Higashiosaka, Japan

**Keywords:** theory of mind, child development, adults, individual differences, executive functioning, language, schizotypy, autism

Theory of mind and its development have been the subject of much research over the last 40 years. Theory of mind is generally thought to be very important in cognitive and social development. However, there is still much debate as to how it should be defined and even as to whether it is a single entity. In particular, there is controversy around the extent to which it should be seen as a specific cognitive function (Gopnik and Astington, 1988; Perner, 1991; Wellman, 2004), or rather as dependent on, or mutually developing with, other cognitive abilities and characteristics, such as language (Tager-Flusberg, 2000; Milligan et al., 2007; Ebert, 2020), metacognition (Kuhn, 2000), executive function (Frye et al., 1995; Carlson and Moses, 2001; Sabbagh et al., 2006; Pellicano, 2010; Devine and Hughes, 2014), and cognitive and perceptual styles that emphasize gist vs. detail (“strong” vs. “weak” central coherence) (Jarrold et al., 2000; Happé and Frith, 2006). It is also possible that theory of mind itself has several different components, which may be related to different degrees of different cognitive abilities and characteristics. Relationships between theory of mind and other cognitive characteristics may also vary with age and may differ between typically developing children and those with autism and other atypical conditions. Gaining a greater understanding of these issues is important to increasing our understanding of theory of mind itself, the nature of cognitive development, the similarities and differences between typically and atypically developing children, and whether it may be possible to devise interventions to improve theory of mind, either directly or by improving other abilities. The goal of the current Research Topic is to bring together articles on various aspects of theory of mind and any concurrent and longitudinal relationships with other cognitive abilities and characteristics.

This Research Topic includes studies of theory of mind in relation to other abilities in children's development, typical adults, and clinical populations. It includes several studies of the relationships between theory of mind and other characteristics in typically developing children. The other characteristics investigated include working memory, vocabulary, fluid intelligence, and various aspects of social competence and understanding. The studies also include discussion of factors other than theory of mind itself which may influence performance in false belief tasks. Children's theory of mind abilities might be underestimated because of the difficulty they experience with the conversational pragmatics of the tasks or overestimated because they may succeed in tasks by reasoning about perceptual access rather than about beliefs.

Wang and Frye investigate young children's concepts of learning and their associations with theory of mind development. In their first study, 75 children between four and six were asked to judge whether characters had learned something. They tended to attribute learning not only to those who had experienced a genuine knowledge change but also sometimes to those who had not but had experienced accidental coincidences. Their performance in this task correlated both with age and with performance in a false belief task. However, after controlling for age, the correlation between performance in the learning attribution task and in the false belief task ceased to be significant. In another study, 72 children between 40 and 90 months were asked to judge whether story characters intended to learn and whether they eventually learned. Children suggested that story characters over-attributed learning intention to situations where learning occurred without explicit intention (discovery learning and implicit learning) and had difficulty with stories where there was a conflict between the learning intention and the outcome. Once again, their performance in the learning attribution task correlated with their false belief task performance, but the correlation ceased to be significant after controlling for age.

Both Baratgin et al. and Pesch et al. investigate the factors that may cause young children to experience difficulty in theory of mind tasks, coming to somewhat different conclusions. Baratgin et al. investigate the possible role of conversational pragmatics in young children's difficulties with the first-order false belief task. The authors point out that being questioned by a presumably knowledgeable adult about “where Maxi will look for the chocolate” might be interpreted as an attempt to test the child's knowledge about the whereabouts of the object, rather than a question about the protagonist's beliefs. They carried out a study of 62 three-year-olds, who were given the task either in its traditional form or where the human adult was replaced by an “ignorant and slow” robot, to whom the child needed to be a mentor. Performance was significantly better in the robot condition than in the human condition, suggesting that the pragmatic difficulty of the standard task may indeed be affecting children's performance.

Pesch et al. argue that children's performance in false belief tasks may involve their reasoning about a protagonist's perceptual access to a set of events, rather than the protagonist's beliefs (Fabricius and Khalil, 2003). They investigated 85 four- and five-year-olds' performance in traditional and modified false belief tasks, true belief tasks, and one component of executive function: working memory. The modified false belief tasks were more complex than the traditional tasks in that they included three or four options rather than just two. Children performed worse in the true belief tasks and the modified false belief tasks than in the traditional false belief tasks. Moreover, when they failed the modified false belief tasks, they were more likely to select irrelevant options than reality options. Performance in the modified tasks was better when they involved contents rather than location, and working memory was related to performance in contents but not location. The authors conclude that their results support the perceptual access theory.

Aspects of theory of mind continue to develop in later childhood. Rosso and Riolfo investigate the performance of 112 middle-grade children in the Reading the Mind in the Eyes Test and test the relationships between performance in this test and age, sex, family characteristics, receptive vocabulary, and fluid intelligence, as measured on the Raven's Matrices. The Reading the Mind in the Eyes Test did not correlate with any family characteristics. It did correlate with both vocabulary and fluid intelligence, but only fluid intelligence turned out to be a significant independent predictor in multiple regression.

O'Grady and Nag conduct a review of 31 studies of typically developing children, mostly of primary school age, who were trained in social cognitive skills. The reviewed studies do not seek to train children in false belief understanding, which tends to reach the ceiling in typically developing children beyond a very young age. The dependent variables in these studies mapped onto the following ToM constructs in at least 87% of studies: “Representation of Others and/or Self,” “Knowledge/Awareness of Mental States,” “Attributions/Explanations of Mental States,” “Social Competence,” “Predicting Behavior,” and “Understanding Complex Social Situations.” The authors propose a hierarchy that organizes these constructs as either skills or competencies within the construct of “Representation of Others and/or Self.”

Individual differences in theory of mind in typical adults are also an important subject. There is no doubt that adults do show significant individual differences in this area, explained in a wide variety of ways by different theorists (e.g., Baron-Cohen et al., 2001; Apperly et al., 2008; Mason and Macrae, 2008; Conway et al., 2019). There has been a significant amount of research into cognitive and personality correlations of such individual differences, but it is investigated in just one study in the present Research Topic.

Török and Kéri investigate relationships between questionnaire measures of mentalization, mindfulness, working memory, and schizotypal personality traits in 300 adults in the general population. They found that, after controlling for mindfulness and working memory, mentalization was negatively correlated with schizotypy and with all its components of unusual experiences, cognitive disorganization, introvertive anhedonia, and impulsive non-conformity. Low mindfulness was an independent predictor of schizotypy, but low working memory was only vaguely related to schizotypy. The authors conclude that weak mentalization is a core feature of schizotypy, independent of mindfulness and working memory.

Several studies in this Research Topic look at people with neurodevelopmental or psychiatric disorders. Autism has been proposed by many researchers over the years, starting with Baron-Cohen et al. (1985), to be closely linked with theory of mind deficits and delays (e.g., Fombonne et al., 1994; Hale and Tager-Flusberg, 2005; Senju et al., 2009; Hoogenhout and Malcolm-Smith, 2017; Altschuler et al., 2018) and, unsurprisingly, features in this Research Topic. Theory of mind abnormalities have also been proposed to be associated with a number of other disorders, including, but not limited to, schizophrenia (e.g., Frith, 1992; Bora et al., 2006), language disorders (Cardillo et al., 2018; Smit et al., 2019), and borderline personality disorder (e.g., Fonagy and Bateman, 2008; Frick et al., 2012; Baez et al., 2015).

Rosello et al. present a study of 52 children with autistic spectrum disorder without intellectual disability and 37 typically developing children. They were given tests on theory of mind and two vocabulary and memory tests. Their mothers answered questionnaires about applied theory of mind abilities, presence and severity of ASD symptoms, adaptive/social skills, and pragmatic competence. A cluster analysis found two groups of children with ASD with “Lower ToM abilities” and “Higher ToM abilities” profiles on all the ToM measures. After controlling for vocabulary and working memory, both groups of children with ASD showed statistically significantly lower applied ToM abilities than the typically developing group. The “Higher ToM abilities” group of children with ASD performed similarly to the typically developing children in the explicit theory of mind task, while the “Lower ToM abilities” group performed significantly worse. The “Lower ToM abilities” group obtained significantly higher scores on autism symptoms and lower scores on adaptive behavior and pragmatic skills than the “Higher ToM abilities” group.

Isaksson et al. investigate theory of mind in autism and other neurodevelopmental disorders within a wider study of cognitive factors that may be associated with theory of mind and genetic and environmental influences on these associations. They carried out a co-twin control study of 311 pairs of twins, 170 of which were monozygotic, with a mean age of 17; 19. There were 134 typically developing pairs and 177 pairs who were concordant or discordant for autism, ADHD, or other neurodevelopmental disorders. They were given the Reading the Mind in the Eyes Test to assess theory of mind, the Fragmented Pictures Test to assess central coherence, The Tower Test to assess executive functioning, and the Wechsler Intelligence Scales to assess IQ. Across pairs, lower IQ and weak central coherence were associated with lower theory of mind performance. Theory of mind performance was higher in older participants and females. It was not associated with executive function. In within-pair analyses, the association between IQ and theory of mind became weaker and the association between central coherence and theory of mind ceased to be significant. This pattern suggests that genetic factors and shared environment may influence the associations between central coherence, IQ, and theory of mind.

Another study looks at dyslexia. Wright and Wright investigate theory of mind in adults with dyslexia, with the aim of studying causal links between linguistic competencies and theory of mind. Dyslexic and non-dyslexic adults were presented with computer-based and non-computer-based vignettes relating to false belief and were asked to answer four types of questions (factual, inference, first-order theory of mind, and second-order theory of mind). In both the computer-based and non-computer-based tasks, dyslexic adults performed worse than non-dyslexic adults in the false belief tasks. However, when given the ToM30Q questionnaire, which makes fewer demands on language and memory, dyslexic and non-dyslexic adults showed no difference in theory of mind. The authors suggest that dyslexic adults, and possibly some other atypical groups, may fail in theory of mind tasks, not because of deficits in theory of mind as such but because of performance limitations caused by weaknesses in language and memory.

Németh et al. assess the executive functions and mentalizing abilities of patients with borderline personality disorder (BPD). Eighteen such patients and 18 healthy controls were tested on IQ, theory of mind (the Reading the Mind in the Eyes Test, and Faux Pas tests), mentalizing about their own emotional states (an alexithymia test), and several domains of executive function. Patients with BPD were impaired compared with controls on the alexithymia measure and the Faux Pas test, but having a BPD diagnosis was a positive predictor of performance in the Reading the Mind in the Eyes Test. Executive functions and IQ predicted performance in the Faux Pas test but were not associated with performance in the Reading the Mind in the Eyes Test or the alexithymia test.

The findings reported in this Research Topic converge in indicating that there are important and quite complex relationships between theory of mind and a wide variety of cognitive characteristics at all ages, in both typically developing individuals and in several developmental and psychiatric disorders. We hope that the findings will inspire yet further research on how such relationships may persist or change with age in both typical and atypical development, the direction of these relationships longitudinally, how they may vary between different disorders, and the extent to which different aspects of theory of mind may show different relationships with other cognitive characteristics. We hope that future studies will provide further insights into whether these relationships differ between typical samples and those with disorders and perhaps thus increase our understanding of whether different disorders should be seen as sharply distinct from typical functioning or as falling on the extreme end of a continuum. We also hope that the findings in this Research Topic, and in future studies that they inspire, may have an impact on the education of typically developing individuals and the treatment of disorders.

## Author contributions

All authors listed have made a substantial, direct, and intellectual contribution to the work and approved it for publication.
